# Sleep in the United States Military

**DOI:** 10.1038/s41386-019-0431-7

**Published:** 2019-06-11

**Authors:** Cameron H. Good, Allison J. Brager, Vincent F. Capaldi, Vincent Mysliwiec

**Affiliations:** 1 Physical Scientist, US Army Research Laboratory, Aberdeen Proving Ground, MD, 21005 USA; 2 Sleep Research Center, Center for Military Psychiatry and Neuroscience, Walter Reed Army Institute of Research, Silver Spring, MD 20910 USA; 3 Department of Behavioral Biology Branch, Center for Military Psychiatry and Neuroscience, Walter Reed Army Institute of Research, Silver Spring, Silver Spring, MD 20910 USA; 4 San Antonio Military Health System, Department of Sleep Medicine, JBSA, Lackland, TX 78234 USA

**Keywords:** Post-traumatic stress disorder, Sleep, Behavioural genetics, Translational research

## Abstract

The military lifestyle often includes continuous operations whether in training or deployed environments. These stressful environments present unique challenges for service members attempting to achieve consolidated, restorative sleep. The significant mental and physical derangements caused by degraded metabolic, cardiovascular, skeletomuscular, and cognitive health often result from insufficient sleep and/or circadian misalignment. Insufficient sleep and resulting fatigue compromises personal safety, mission success, and even national security. In the long-term, chronic insufficient sleep and circadian rhythm disorders have been associated with other sleep disorders (e.g., insomnia, obstructive sleep apnea, and parasomnias). Other physiologic and psychologic diagnoses such as post-traumatic stress disorder, cardiovascular disease, and dementia have also been associated with chronic, insufficient sleep. Increased co-morbidity and mortality are compounded by traumatic brain injury resulting from blunt trauma, blast exposure, and highly physically demanding tasks under load. We present the current state of science in human and animal models specific to service members during- and post-military career. We focus on mission requirements of night shift work, sustained operations, and rapid re-entrainment to time zones. We then propose targeted pharmacological and non-pharmacological countermeasures to optimize performance that are mission- and symptom-specific. We recognize a critical gap in research involving service members, but provide tailored interventions for military health care providers based on the large body of research in health care and public service workers.

## Introduction

Sleep must be properly calibrated and carefully considered during nearly every aspect of one’s military career. Service members are notorious for sleeping at any given opportunity, and often fall asleep quickly in non-traditional, noisy environments. Achieving adequate amounts of restorative sleep is an ongoing problem that is critical for reasons of personal safety, unit performance, and even a matter of national security. Although there are educational resources in place for optimizing sleep in military personnel (e.g., Army Office of the Surgeon General’s Performance Triad (P3) initiative), poor sleep hygiene in the long-term can increase sleep problems co-morbid with physiological and psychological issues that include: cardiovascular disease [1], substance abuse [2], post-traumatic stress disorder (PTSD), and mood disorders [3–5].

This review is written at a time when US military leaders are giving increased attention to the critical importance of sleep for next-day performance and long-term physical and mental health across a military career. For instance, the US Army’s P3 highlights that adequate sleep, physical activity and nutrition are essential for increasing and sustaining mental and physical performance. The US Army Field Manual (FM) 6–22.5 further recognizes the importance of sleep and provides guidance on how to implement preventative measures for decreasing sleep disturbances, as well as providing military leaders direction on how to cope and counterbalance periods of insufficient sleep/disrupted sleep due to operational contingencies through evidence-based pharmacological and non-pharmacological treatment strategies.

Here, we cover results from both clinical and preclinical studies, highlighting military relevant literature where possible. A majority of military-focused studies on the impact of sleep on next-day operating performance are limited to self-report and/or only capture impact pre- and post- deployment or at a single time point. Fortunately, there are numerous human studies of cognitive, endocrine, and molecular function during reverse sleep cycles (i.e., night shift work), 24 h work schedules, and rotating (rapidly shifting) work schedules in healthcare (e.g. nurses) and public service (e.g., firefighters) workers with translational value to military personnel. Military-focused studies on sleep disorders are comprehensive, and capture problems across a single deployment (pre- and post), re-deployment, and biological sex. Areas where preclinical animal research can be improved or tailored for military relevance are also emphasized. Lastly, we examine new tools developed by defense laboratories that provide timely performance predictions (2B-Alert), coupled with potential countermeasures and techniques, to combat fatigue during sleep loss in the operating environment.

### Overview of sleep

Sleep wake states can be classified as waking, rapid eye movement (REM) and non-rapid eye movement (NREM) sleep based on characteristics of electroencephalography (EEG) and electromyography (EMG) signals. Waking is defined by high-frequency, lower amplitude EEG signals and a robust EMG signal, owing to active postural and kinetic muscle activity. In contrast, NREM sleep EEG exhibits lower frequency, higher amplitude signals following more synchronized neural firing patterns and a reduced amplitude EMG. REM sleep EEG reverts back to high-frequency, lower amplitude signals reminiscent of waking, yet the EMG signals are devoid of activity, referred to as atonia, which is used to differentiate REM from waking in polysomnographic recordings [6].

Sleep and waking are both active processes in the brain, yet different mechanisms drive each state. Cognitive arousal is largely mediated by excitatory brainstem structures and neurochemicals such as the peduncolopontine nucleus (acetylcholine), locus coeruleus (norepinephrine) and raphe (serotonin), as well as the midbrain tuberomamillary nucleus (histamine) that activate thalamic and cortical structures. In contrast, inhibitory cells in the hypothalamus (ventrolateral preoptic area) send γ-aminobutyric acid (GABA) and galanin projections to inhibit these structures, amongst others, to initiate sleep. Recent evidence from another midbrain inhibitory structure, the rostromedial tegmental nucleus [7], suggests a distributed ventral inhibitory system may exist that promotes sleep (Jhou and Good et al., unpublished data) [8]. One explanation of this reciprocal relationship is referred to the “flip-flop circuit” theory proposed by Saper et al. to finitely explain hypothalamic regulation of NREM sleep and NREM transitions to REM and waking states [9, 10].

During development, sleep/wake patterns change such that infants require approximately twice the amount of sleep time as mature adults, but do so in a fragmented pattern throughout the day and night [11–13]. As development progresses, the number of sleep bouts gradually consolidate to the adult cycle consisting of a single ~8 h nightly sleep episode during which the individual transitions through NREM and REM stages. In adolescents and early adulthood there is an increase in sleep pressure that can escalate sleep need and duration to ~9 h per night [14, 15], and shift bedtimes and awakenings to later hours. This time period coincides with the age group who typically enlists in the United States (US) military post-high school (17–20 years of age) [16]. At this ontogenetic time, sleep/wake cycles are still transitioning to adult patterns [11]. This runs counter to the sleep/wake schedules of military training to which these adolescents enter, where <6 h of sleep per night and rise times of 04:30 are commonplace [17–20]. Unfortunately, insufficient sleep <6 h per night becomes chronic and normative during early training and is pervasive even at elite military academies [17, 19]. To test the impact of sleep schedules on health and performance, Miller et al. shifted the sleep schedules of US Army trainees during Basic Combat Training to better align with the natural sleep drive and habits of adolescents. Results showed reductions in mood disturbances, improvements in marksmanship and less fatigue, as well as overall improvements in scores of sleep quality [18], highlighting the significant impact of natural sleep drive on human performance. It’s hypothesized that these early adolescent improvements in sleep hygiene during basic training, a time when most trainees sleep patterns are still developing, could carry forward to increased resiliency against future circadian/sleep disruptions or mental illness. Longitudinal studies suggest that sleep disturbances in young adults serve as an early risk factor for developing major depression [21–23], and this risk could persevere for many years [24]. Additionally, pre-deployment insomnia is a significant contributor to post-deployment PTSD and suicidal ideation [25–27]. A recent model of insomnia in US military veterans identifies early sleep problems as an initial, precipitating stressor that may reduce resilience to subsequent stressors, including persistent sleep problems [28]. This feed-forward pattern could impair an individual’s adaptive capacity to cope with additional or larger stressful events, predisposing them to depression, anxiety or PTSD. Whereas it remains unknown if mitigating sleep disturbances early during basic training could afford protection against future mental illness in military populations, effort should be directed to determining if early intervention to minimize sleep debt could reduce subsequent negative outcomes in this susceptible population. Although, given the military culture where sleeping less is common throughout ones career, and is largely viewed as a sign of mental and physical toughness, it is unclear if early improvements in sleep hygiene could overcome many subsequent years of disordered sleep.

Nightly sleep duration for US service members is truncated, as compared to civilian counterparts. The National Sleep Foundation, American Academy of Sleep Medicine, and Sleep Research Society all recommend a minimum of 7 h of sleep per night for adults 18 years of age or older [15, 29]. Indeed, a large national US epidemiology study found that 63% of Americans slept 7–8 h per night, whereas only 28% slept 6 or fewer hours per night, consistent with short sleep duration (SSD) [30]. This contrasts with two US military studies which found 72 and 69% of service members were classified as SSD with less than 6 h of sleep per night, and only 27 and 30% obtained the recommended 7–8 h of sleep, respectively [3, 31]. Redeployed US Army Soldiers with prior combat exposures were most likely to have SSD, whereas being wounded or injured during combat was a strong predictor of sleeping less than 5 h per night post-injury [31]. Operationally, SSD truncates opportunities to maximize the recuperative value of sleep required of highly mentally and physically demanding tasks inherent of military operations. SSD also degrades next-day performance as found in a sleep duration study of artillery marksmanship [32, 33], compromising personal safety as well as resources.

### Consequences of military work schedules

The biological impact of rotating work schedules has been extensively studied, yet examples focused on military personnel and operations are sparse despite operational work cycles of 12 h and 24 h being common-place. This is due in part to the non-controlled dynamic settings in which data would have to be collected, where the mission comes first and duties cannot be routinely interrupted for data collection efforts. Even prospective studies evaluating the effects of shift schedules are challenging in the military population due to the variable and temporary nature of most work schedules and environments, and are more likely to yield less conclusive results than civilian studies where work schedules tend to be more consistent. Further, the confounding and variable factors that must be statistically accounted for across subjects would be immense given the situation and task specific nature of service member’s duties. Fortunately, there are numerous human studies of cognitive, endocrine, and molecular function during reverse sleep cycles (i.e., night shift work), 24 h work schedules, and rotating (rapidly shifting) work schedules in healthcare (e.g. nurses) and public service (e.g., firefighters) workers with translational value to military personnel.

One military population that is more conducive to short-term studies of shift work is the Navy, where Sailors are deployed on ships for days to weeks at a time and tend to have more defined watch duties while at sea. Researchers located at the Naval Postgraduate School have published a number of studies that leveraged this population, with particular emphasis on deriving watch schedules that better align with circadian rhythms [34] and offer more time for dedicated sleep [35] than the common rotating 5 h on/10 h off watch schedule [20]. These studies consistently suggest that Sailors prefer the 3 h on/ 9 h off watch schedule, with less reported daytime sleepiness and improved mood and reaction times with fewer errors. Although this military population is more amenable to studying the effects of shift schedules, care still must be taken when generalizing sleep and physiology results across ship departments [36, 37]. For instance, salivary cortisol levels can vary across individuals due to increased stress in noisy departments (e.g., engine rooms and gun turrets), whereas light intensity can shift melatonin levels depending on duty station (e.g., interior control room versus outdoor ship bridge) [37]. In another military population, surveyed US Army Aviation personnel working a reverse sleep cycle reported that they did not achieve adequate daytime sleep when working this schedule [38], which can lead to pilot errors [39].

#### Night shift work (reverse sleep cycles)

In general, night shift work of any kind and duration increases morbidity and mortality, negatively impacting both physiological and psychological health. Regarding physiological health, night shift work in female nurses leads to unhealthy lifestyles that excludes exercise [40] and increases overall caloric intake and craving for high-fat, high-sugar, and protein-deficient foods [41] by means of altering gut-derived release of hormones regulating hunger and satiety (ghrelin) [42]. Interestingly, positive stress such as exercise has even been shown to exacerbate clinically significant endocrine disruption of ghrelin, leptin, insulin, and triglyceride levels [43] induced by night shift work [44, 45], indicating that the timing of exercise must also be properly calibrated in night shift workers. These results have direct corollaries with military populations, leading to reduced ability to control one’s weight despite semi-annual evaluations in order to maintain current military occupation and to determine odds of deployability (AR 600-9). In the long-term, a higher body mass index (BMI) “set point” in service members manifest from shift work may lead to gastrointestinal tract issues and type 2 diabetes as found in civilian counterparts [40, 46, 47]. Night shift work can also reduce skeletomuscular (isometric) strength by 20% in civilian populations [48], can contribute to increases in musculoskeletal symptoms in US Navy crewmembers [49], and lead to cardiovascular stress through an increase of the blood-borne marker, cyclooxygenase-2 (COX-2) [50]. As a longitudinal consequence of night shift work (20,142 shift workers studied across 22 years), new cases of hypertension increased by >10% [51]. This trifecta of hypertension, reduced isomeric strength, and higher BMI increase the risk for cardiovascular disease. Finally, night shift work in police officers – a civilian occupation with many overlapping responsibilities as military personnel – have heightened blood-borne markers of inflammation (increased white blood cell counts, lymphocytes, and monocytes) [52], potentially leading to new cancer cases specific to biological sex (breast [female] and prostate [male]). In the short-term for military personnel, this trifecta could increase the risk of musculoskeletal injuries [49] and/or myocardial infarction while engaging in physically demanding tasks specific to military occupation. Post-military service, this trifecta could contribute to the increased risk for heart disease in veterans [53]; a finding that could be compounded by the lack of mandatory early morning exercise sessions and field training that are no longer part of a service members routine, as well as injuries or neurological disorders incurred during service, amongst other factors.

Regarding psychological health and performance, basic and advanced cognitive processes such as reaction time to respond to visual cues and the ability to quickly and correctly perform mental calculations are impaired in nighttime healthcare workers compared to daytime counterparts [54, 55]. Similar to healthcare workers, military occupations have unique skill sets requiring service members to accurately and rapidly attend to, process and integrate new information specific to one’s occupational duties as part of a military mission. Cognitive shortfalls in the ability to quickly learn, remember, and execute a specific task can lead to mission failure and can compromise individual and unit safety as well as national security. Further, night shift work leads to long-term elevations in psychological stress and “burnout” that can be detrimental to sustained performance [56]. The extent of reduced attention and vigilance during night shift work can be predicted by, and is directly related to, the extent of misalignment between working schedules and two distinct physiological processes: (a) daytime peaks in core body temperature (when alertness is higher); and (b) nighttime peaks in dim-light melatonin onset (DLMO; when alertness is lower) [57].

Two separate population-based cohorts derived from the Swedish Twin Registry (largest twin registry in the world) demonstrated a clinically significant, dose-response relationship between duration of shift work and night shift work (in years) and incident risk for neurocognitive disorder (as determined from International Classification of Disease [ICD] and Anatomical Therapeutic Chemical Classification System [ATC] codes from patient registers) [58]. This risk was amplified in carriers of the APOE4 mutation [58], common in individuals of European descent [59], who worked a shift or night schedule for more than 20 years. Results from this study suggest that shift work of any duration could increase the risk for neurocognitive disorder many years later, although this needs to be confirmed in other populations. Similar studies should be explored in military veterans, where medical histories, occupation, and work histories, and deployments are documented. The interplay between shift work, night shift work, and traumatic brain injury is another avenue that could yield insight into increased risk for subsequent neurocognitive disorders.

#### Rapid time-zone, light/dark shifts

In general, light is the most potent environmental stimulus capable of entraining, but also phase-shifting, the mammalian sleep/wake cycle. Depending on when white- or blue-enriched light (but not red-enriched light) of any duration is presented at night, light will phase-delay or advance the sleep/wake cycle, as characterized by the photic phase-response curve [60, 61]. This physiological response to timed light exposure can help ameliorate the negative consequences of night shift work as first shown >20 years ago [62]. Light timing and quality does matter as evidenced by the implementation of light “recipes” [63–65] and the efficacy of light-block wearables for optimizing immediate physiological alertness under various night shift schedules [66]. For military personnel, timed light “recipes” can be a solution for phase-shifting sleep/wake cycles for the purpose of “owning the night,” and executing a successful night mission that maximizes performance and reduces risk for injury.

Poor quality and limited artificial incandescent light is common of military installations with 24 h operation centers, necessitating an understanding of the short-term and long-term impact of these conditions on physiological and psychological health in military populations. Incandescent lighting compared to natural sunlight (>10,000 lux) was shown to reduce sleep quality through a dampening in the amplitude of nighttime melatonin release [67], and natural light compared to artificial light results in better consolidation of a sleep/wake cycle through more robust release of brain-derived melatonin [67]. Rapid changes in sleep/wake patterns can shift melatonin release out-of-phase with military duty schedules and result in increased sleepiness during work hours when vigilance needs are high [37]. Whereas a constant dim light routine typically yields benign impact on physiological and psychological health, chronic bright light was shown to cause sleep/wake arrhythmia and sleep/wake rhythm splitting [68], dampen rhythmic gene expression [69], and functionally lead to reduced insulin sensitivity and immune deficiency in rodent models [70, 71]. Nighttime light pollution is also a significant problem in operating environments. Nearly all service members who have to relieve bodily functions in the middle of the night have to get dressed and walk more than 250 m outside in order to use the latrines [39], which can be located inside a facility that is lit 24 h using incandescent lighting. Stadium-type lighting is also used to illuminate these trekked paths on operating bases, further compounding light pollution in already poor living quarters for restorative sleep. In both instances, the bright light can serve as a zeitgeber that reduces an individual’s ability to fall back asleep upon returning to their bunk, further dysregulating their sleep patterns. With better integration of more natural lighting on military installations, service members could maximize their opportunities for achieving restorative sleep on non-training days and, most importantly, to maximize this opportunity for “sleep banking” leading up to sleep loss during multi-day training exercises or operations [72].

Sub-acute physical and psychological stress can act as non-photic zeitgebers in rodent models by means of altering circadian-controlled rhythms of corticosterone [73, 74]. Physiological stress reactivity in humans is circadian-controlled [75], suggesting that stressors may potentially alter circadian rhythms at specific times of the day. It is well known that service members are exposed to more acute physiological and psychological stress in operating environments compared to the general population. Therefore, amplitudinal and phase-shifts in cortisol rhythms induced by acute stressors experienced by service members may also serve as a zeitgeber [37], similar to bright light exposure, but certainly warrants further investigation. Nonphotic stimuli such as exercise [76–79] and social interaction [80] are also capable of entraining and phase-shifting the mammalian sleep/wake cycle, [78, 79, 81, 82], yet nonphotic cues are highly complex and can cancel out the phase-shifting actions of light in animal models [81]. In humans, exercise can phase-shift circadian rhythms; an effect that is additive to bright light exposure [83]. Time of day also plays a role, with recent evidence suggesting multiple time windows to include exercise to adjust circadian rhythms [84]. These exciting results suggest the timing of novel non-photic stimuli (e.g., military warrior tasks and battle drills) must be carefully controlled when seeking to purposefully and effectively phase-shift a sleep/wake cycle with enriched light for greater performance operability at night. Although, this level of circadian coordination is not currently adopted by military units.

To add to the problem, around-the-clock military operations often result in routine back-to-back phase-advances and subsequent phase-delays of traveling from “safety bases” >2 time zones away from the front line of troops. Recent research in an animal model demonstrated that phase-advances and subsequent phase-delays (or vice versa) can be a “zero sum game” by means of creating circadian decoupling and dampening rhythmic gene expression [85] and functionally impairing memory encoding and recall [86, 87]. Therefore, there is a critical need to better understand these biological underpinnings of human performance in response to novel photic/non-photic stimuli specifically for military personnel in order to better operate at night, while also ensuring that the physiological and psychological health of military personnel are being optimized in the short- (combat) and long-term (career and retirement).

#### 24 h operational tempos

In contrast to the majority of civilian occupations, US military operations are around-the-clock. Unpredictable and ever changing duty schedules and >24 h operations are not only common during deployment to a combat zone (i.e., operating environment), but are also seen stateside (i.e., in garrison) in order to create a continuous information flow to and from the operating environment. Service members must often engage in mid-night teleconferences with a unit operating >8 time zones away in the Middle East or Pacific Islands (anti-phasic coordination), and then still perform their daily duties the next day under shortened and fragmented sleep. The reverse is also common; higher military headquarters communicating during daylight hours in Washington, DC with units engaging in nighttime operations in the Middle East often means that service members executing nighttime operations are awake >12 h preparing for the mission even prior to the overnight mission itself. This scenario is exemplified by the US military’s pride in “owning the night,” meaning that high-risk operations against enemy forces are prioritized at night instead of during the day (e.g., mid-night capture of Osama bin Laden in Abbottabad, Pakistan in 2011). Although we, as a nation, will also often remember and glorify these highly publicized overnight missions, many smaller scale and lesser publicized nighttime missions have significantly compromised the health and safety of US service members and have resulted in mission failure as a consequence of long-standing nighttime shift work, coupled with chronic insufficient sleep, that affords many opportunities for performance decrements and errors following this operational schedule. Outside of combat operations, it is also very common for service members to go on multi-day training exercises and sleep <5 h per night (Skeiky et al., unpublished data). Thus, around-the-clock military operations continue to place added risk on life-and-death decisions made by sleep-deprived service members working in highly stressful environments across many geographical regions and battlefield domains (i.e., land, sea, and air) with increasingly advanced weapon and data systems requiring a high degree of cognitive throughput. Given the requirement to complete physically and mentally demanding tasks on a consistent basis during mission execution, the inability to perform and recover due to the inability to achieve restorative sleep, is a setup for mission failure.

Studies examining the consequences of a single night of >24 h sleep deprivation and chronic sleep restriction (<7 days) have yielded immediate, short-term compromises in physiological and brain health at levels of neural connectivity, endocrine and molecular signaling cascades, and psychological/behavioral outcomes. For instance, a study of real-time brain function via positron-emission tomography (PET) found that a single night of >24 h sleep deprivation rapidly increased tau- and amyloid plaque accumulation – two gold-standard signatures of brain inflammation and risk for Alzheimer’s disease – that did not fully return to baseline levels after recovery sleep [88]. Immediate, clinically significant declines in anabolic processes such as decreases in blood-borne testosterone and growth hormone release [89, 90], concurrent with increases in catabolic processes such as blood-borne cortisol release [89–93] and oxidative stress [94] have been reported following acute sleep deprivation. Extended sleep deprivation also phase-shifts and dampens the amplitude of biochemical substrates of glucose, fatty acid, and amino acid metabolism: including cholesterol [95], acylcarnitines [96], oxalic acid [97, 98], and diacylglycerol [97, 98]. At the most microscopic, cellular levels of study, sleep deprivation alters the human DNA transcriptome [99] and ATP synthesis [94]. These human studies show the broad neural, endocrine, tissue, and cellular impacts of a single night of >24 h sleep deprivation and chronic sleep restriction (<7 days) on both physiological and psychological health that are highly relevant to the duties of service members.

### Sleep and psychiatric disorders

It is estimated that over 60% of service members who never, previously, or were currently deployed sleep less than 6 h per night, with those previously or currently deployed more likely to report sleeping <5 h per night [100]. There are several factors contributing to the lack of sufficient sleep in the military, including combat operations, shift work, and the comorbidity of psychiatric disorders in service members. Although real-time physiological and psychological health data in active duty personnel during deployed military operations is sparse, sleep disturbances, namely insomnia and obstructive sleep apnea (OSA), have been broadly studied pre- and post-deployment and across sexes in medical treatment facilities [3–5, 101, 102]. Insomnia is often a principal symptom in many psychiatric disorders, most notably in PTSD, depression, and mild traumatic brain injury (mTBI). In a study of service members with combat-related head injuries, 55.2% experienced insomnia symptoms, and 90.5% had at least one comorbid psychiatric condition [103]. Whereas sleep disturbances may result from psychiatric injury, pre-morbid disordered sleep may also predispose a service member to developing PTSD and other psychiatric conditions [104].

**Fig. 1:**
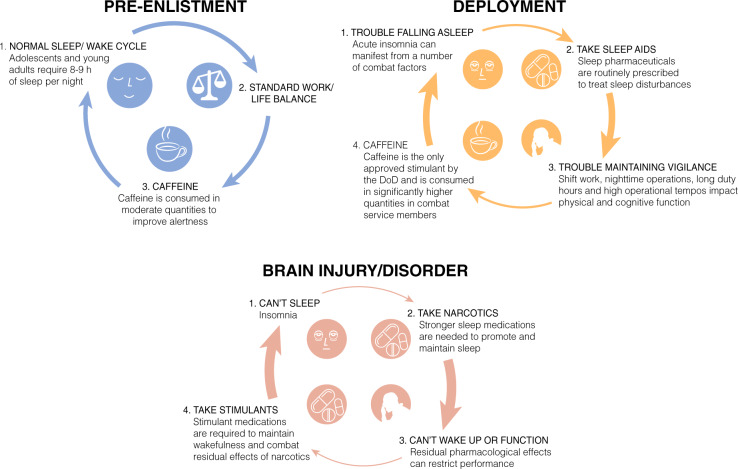
Three sleep/wake scenarios are represented based on discussion throughout the manuscript. 1. As illustrated in blue, enlistees generally join the military with normal sleep/wake habits that must rapidly transition to an environment where restricted and fragmented sleep is the norm. 2. Upon deployment, as shown in orange, a number of precipitating factors can cause acute insomnia that require prescription sleep aids to help service members fall asleep. Residual pharmaceutical effects, combined with long duty hours and irregular sleep schedules, result in statistically higher amounts of caffeine intake to maintain alertness and performance. If taken at the wrong time, the awake-promoting effects of caffeine can cause further sleep disruptions and prevent restorative sleep during an already limited sleep window. This feed-forward cycle can necessitate service members being prescribed an increasingly potent sleep aid to overcome the stimulant effects, further necessitating stimulants to transition to wakefulness and maintain vigilance throughout subsequent duty hours, as illustrated by the increasing thickness of the arrows in the cycle. 3. Following injury or diagnosis of neurological disorder, as shown in red, persistent insomnia occurs in a large number of service members. Stronger narcotic sleep aids are routinely prescribed to overcome insomnia, creating a stronger dependence on pharmacology to drive sleep/wake states, as depicted by the even thicker red arrows within the cycle

Comprehensive reviews have been written on the integral correlations between sleep and traumatic brain injury (TBI) [105, 106] and PTSD [3, 100, 107, 108], including a review by Neylan et al. appearing in this volume. Although discussing the surrounding literature is outside the scope of the current article, it should be noted that the majority of service members that endorse PTSD will also endorse significant insomnia or other sleep disturbances, (i.e. nightmares), posing a unique challenge to treating this patient population (Fig. 1).

#### Insomnia

Insomnia is the inability to fall asleep or stay asleep resulting in impaired daytime function and subjective patient distress. Generally, insomnia is characterized as either transient (lasting less than a month) or chronic (>1 month). The first step in treating insomnia is identification of the underlying cause or causes contributing to the patient’s complaint, as often a symptom of an underlying psychiatric condition: PTSD, depression, generalized anxiety disorder, panic disorder, bipolar disorder, and substance dependence. In fact, several studies of active duty military personnel (ADMP) referred to a sleep clinic had insomnia co-morbid with chronic pain, PTSD, anxiety and/or depression [3–5].

Military providers screen for mood, anxiety, and substance use disorders prior to referral to a sleep specialist [109]. The causes for patients presenting with transient or short-term insomnia are more easily identified than patients with chronic insomnia. A service member may experience insomnia at the beginning of a deployment because of changes in their sleeping environment, excessive noise, jet lag, shift work, the stress of being separated from their families, unpleasant room temperature, or anxiety about death or injury during deployment [110]. For instance, a cross-sectional study of deployed US Air Force Airmen found 40% of respondents had a sleep efficiency of <85% or extended sleep-onset latency >30 minutes, whereas 75% reported diminished sleep quality as compared to at home [39]. Further, night shift workers were statistically more likely to have lower sleep efficiency, greater sleep latencies, and report disturbed sleep during daylight hours due to loud noises in their surroundings. Overuse of psychostimulants such as caffeine or modafinil prescribed for shift work can further disrupt sleep patterns, and can create a feed-forward pattern where more caffeine or prescription medication is required to maintain vigilance during the day, despite the negative consequences it has on falling asleep that night (Fig. 1). This cycle is confounded by the additional stressors one endures during deployment, as discussed above. Unfortunately, insomnia often does not resolve when the service member leaves a combat zone. Difficulties initiating and maintaining sleep often persist for months after a service member returns home. Other causes of insomnia for the service member include:

### Sleep state misperception

- This condition is characterized by the patient perceiving that they are not getting enough sleep without objective evidence (i.e., polysomnography) [3, 111, 112].

### Inadequate sleep hygiene

- Patients engage in non-sleep-promoting activities such as exercise or use of alcohol or stimulants (i.e., caffeinated beverages) prior to bedtime [84, 113–119].

### Altitude Insomnia

- Acute adjustment to high altitude (>4,000 feet above sea level) can contribute to insomnia and increased daytime somnolence due to the stimulation of peripheral chemoreceptors [120–123].

### General medical disorders

- Disorders such as congestive heart failure, chronic obstructive pulmonary disease, peptic ulcer disease, pain, and gastroesophageal reflux disease are just a few general medical conditions that may contribute to insomnia [124–128].

### Neurologic disorders

- Neurologic conditions such as strokes, TBI, headache syndromes (i.e., migraine and cluster headache), trigeminal neuralgia, and neurodegenerative disorders. (i.e., Alzheimer’s disease and Parkinson’s disease) all can contribute to insomnia and change sleep architecture [27, 129, 130].

**Fig. 2:**
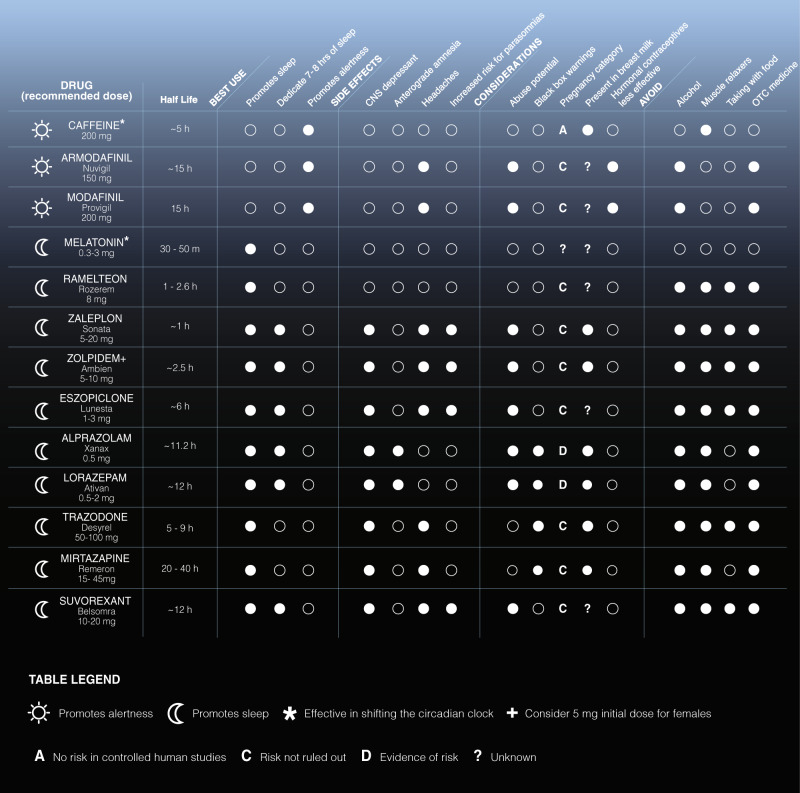
List of common wake (sun) and sleep (moon) medications prescribed to military personnel; all Food and Drug Administration (FDA) approved medications are available for prescription at military treatment facilities. In general, over-the-counter drugs (OTC; e.g. melatonin) are preferred first, followed by non-benzodiazepines. Benzodiazepines are least preferred given their pronounced side effects and deleterious impact on function and performance. Use of trademarked names does not imply endorsement by the U.S. Government and is intended only to assist in identification of specific medications

The most effective treatments for chronic insomnia are non-pharmacological, such as cognitive-behavioral therapy (individual and group) [110, 131–133], as well as motivational interviewing and mobile health delivery of sleep recommendations [134]. Service members are also asked to complete a sleep diary during treatment for insomnia and complete education about sleep hygiene and stimulus control measures. Despite this, use of sleep pharmaceuticals by military personnel is very high. In 2018, we found that nearly half of active duty Army personnel (>100,000 service members) were prescribed zolpidem (trade name: Ambien) or eszopiclone (trade name: Lunesta) as part of a deployment or routine visit to a military treatment facility for sleep complaints (Devine et al., unpublished data). Sleep pharmaceuticals largely target inhibitory pathways through GABA signaling, resulting in reduced short-term and long-term neurobehavioral performance in attention and reaction time that can manifest in a dose-dependent manner [135, 136]. A large list of sleep/wake medications approved for use by military personnel are found in Fig. 2 (half-lives and side effects derived from a Department of Defense formulary search tool). In lieu of residual, inhibitory side effects and increased sleep inertia common of sleep pharmaceuticals, the trade-off is that military personnel must have a high level of personal accountability for use. Missions require service members to be “on call” for 24 h, especially in combat zones. A service member who used a sleep medication the night prior to a planned mission in the early morning or an unplanned mission after mid-night awakening would likely have reduced combat effectiveness and compromised safety. As such, US Army guidelines (ATP 6–22.5) dictate that a Soldier have at least 8 h of non-work time after taking a prescription sleep aid, although it is unknown how well this is followed.

To date, one of the most comprehensive studies of sleep amounts, disrupted sleep, and co-morbidity with physiological and psychological health states and persistent sleep disturbances in military personnel comes from the Millennium Cohort Study (MCS) [137, 138]. MCS was a 7-year study of self-reported physiological and psychological health pre- and post-deployment and across multiple deployments in >55,000 service members from all US military service branches who were active duty, Reserve, or National Guard. 22% of this study population were deployed in support of Iraq and Afghanistan. MCS found co-morbidity of insomnia symptoms with lower self-rated health, more lost work days, lower odds of deployment, higher odds of early discharge from military service, and more health care utilization [138]. Co-morbidity was highest for “short sleepers” (<6 h per night) and “long sleepers” (>8 h per night), showing intra-individual variability in a service member’s sleep “homeostat” (set point). Although, the co-morbidity with long sleepers could be explained by an underlying mood disorder (e.g. depression) that was not fully captured by the questionnaires [138]. Further, the MCS determined that sleep duration and insomnia symptoms pre-deployment were risk factors for new-onset mental health disorders following military deployment [25]. Finally, a separate study determined that the prevalence (and impact) of short sleep duration persisted during re-deployment to Iraq and Afghanistan [31].

#### Obstructive sleep apnea

OSA is pervasive in ADMP [5, 101, 102], similar to studies of OSA prevalence and severity with respect to biological sex in civilian populations [139–141]. However, salient differences between the patient populations of civilian studies [139–142], as compared to ADMP studies [5, 102], exist. Namely, ADMP diagnosed with OSA are younger (<35 compared to >40 years of age), have lower BMI (<28 compared to >30), and are more physically active than civilian counterparts; age, BMI, and physical activity are three key predictors of risk for OSA, in general. Greater than 30% of ADMP who present with a sleep disturbance have insomnia co-morbid with OSA [5, 101, 102]. Of greatest concern in the OSA studies in ADMP is the high degree of co-morbidity of insomnia and OSA with other physiological (chronic pain) and psychological (anxiety and depression) health states, specifically for women [5, 102].

Although there are far fewer female service members compared to males, recent US government regulations now allow females to serve as combat arms officers and in forward operating units. Whereas studies of insomnia co-morbid with OSA in ADMP do not show any sex differences in self-reported sleepiness and insomnia [5, 102], polysomnography revealed a two-fold longer sleep-onset latency for women with an insomnia-OSA diagnosis and differential time spent in light NREM and REM sleep compared to males [5, 102]. In addition, female service members with a sleep disturbance had greater co-morbidity of an insomnia-OSA diagnosis with anxiety/depression not seen in male counterparts [5, 102]. Of the 100 ADMP females from Capener et al. who had a sleep evaluation, 35% had a diagnosis of insomnia-OSA (compared to 37% with insomnia and 15% with OSA, only) that was additionally co-morbid with pain at a rate of 59%, 49% for anxiety, 47% for depression, and 22% for PTSD. Thus, sex differences in the co-morbidity of insomnia-OSA with physiological and psychological health will continue to be pervasive and a critical decision point for the military as the rate of females serving in forward operating units continues to rise.

### Military relevant animal models

Recapitulating the military sleep environment, particularly a combat scenario, in the laboratory using rodents is a challenging endeavor. Imagine a scenario where a service member has been deployed to a combat zone where they’ve been consistently operating on little sleep, varied operational schedules, and are under heavy stress daily. Five months after initial deployment and while on dismounted patrol, a large improvised explosive device (IED) detonates nearby, resulting in TBI to the service member. To understand the human impact of restricted sleep on TBI symptoms or severity, long-term prospective studies would be required to routinely monitor a large number of troops throughout deployment for clinical sleep disorders and exposure to other risk factors, followed by a diagnosis of TBI or PTSD, for example. Rodent studies offer an option to accelerate these studies and control test variables and treatment options. Yet, how can we accurately model this combat scenario, or any number like it, in the laboratory? On one hand, we have to develop and validate paradigms of longer-term and complete sleep cycle disruption that better model operating environments. We then must merge these paradigms with relevant models of blast, impact or stress to induce TBI or PTSD-like symptoms in these animals, acknowledging that each aspect of these models in of themselves are likely to result in physiological and psychological changes to the animals that must be understood and placed in the broader context of human sleep conditions. How the two manipulations interact is unknown, but should be emphasized in future studies.

Lower mammalian species permit invasive studies on the neural underpinning of sleep and sleep disruption that are not possible using human subjects. Rodents have inherently different sleep/wake patterns than humans (fragmented versus consolidated sleep/wake patterns, respectively), yet certain features of the sleep/wake system have been conserved across species that permit results to be translated. At present, the laboratory mouse (*Mus musculus*) has become the most prevalent rodent model for studying sleep/wake neurocircuitry due in part to conserved genetic regulation of sleep/wake states and sleep/circadian rhythm disorders that are similar to humans, including:

- baseline (normal) sleep amounts and homeostatic responsiveness to sleep loss (Bmal1) [143] [mouse]; [144][mouse]; [145] [human]);
- sleep timing (Per2: [146] [mouse]; [147] [human];
- short sleepers (Dec2: [148] [mouse]; [148] [human]);
- sex-linked regulation of sleep/wake processes (Ube3a: [149] [mouse]; [150] [human]; Sry: (Brager et al., unpublished data), [mouse]; and
- broad quantitative trait loci studies in mice [151] and equivalent genome wide studies in humans [152, 153].

Further, widespread integration of genetic tools and techniques (e.g. optogenetics) now allow cell type and pathway specific studies into the brain regions that control sleep/wake states [154–157]. This is a critical advance, as early research into the neuroanatomical basis of these states relied on gross surgical techniques to sever pathways, as well as electrolytic or chemolytic ablation of a brain area (rodent), or as a consequence of stroke or injury (humans) to isolate and assess the influence of distinct brain regions.

#### Sleep as a metric in military relevant animal models

Environmental circadian disruption (ECD) in animal models, achieved through routine phase-shifts in the light-dark cycle, is of great relevance to better understand the consequences of military operations on physiological and psychological health. In rodents, regimens of ECD have residual impact on total sleep time and amplify misalignment of activity rhythms with sleep rhythms [158]. The consequence of this misalignment is a heightened pro-inflammatory response exclusively due to the phase-shift in the light-dark cycle, rather than concurrent sleep loss [158]. Regimens of ECD also impact metabolic processes on whole-body, macroscopic [159] and microscopic scales (heart [160]; skeletal muscle [161]; liver [162]) similar to what has been reported in human models of sleep loss compounded by shift work [54, 163, 164]. Animal models of constant light and constant darkness can desynchronize the suprachiasmatic nucleus (SCN) and alter gene activity [68, 69], and can further our understanding of the consequences of extended shift work and night operations in military personnel.

Whereas TBI research utilizes a variety of tools and techniques to simulate an injurious event to the brain [165], compressed air driven shock (blast) tubes are routinely used to simulate a concussive event like the one described above [166]. In a generic shock tube experiment, animals are removed from their home cages, anesthetized, and placed in a restraint device at the end of the tube. Following a shockwave event of set magnitude, the animals are removed from the tube and generally treated in some manner in an effort to mitigate the effects of shock loading. Behavioral assays are run days to weeks later to evaluate treatment efficacy, followed by neuropathology assays of neural tissue to quantify cell damage [167]. A key missing variable from this study design is the effect of sleep deprivation prior to the blast event, or how the simulated blast event disrupts sleep patterns post-blast. This is despite sleep disorders occurring in a high percentage of deployed military personnel [3], as well as clinical sleep disorders being a common symptom in TBI [105, 168–170].

Relatively few animal studies have investigated the effect of TBI on post-injury sleep, and to our knowledge none have used the military relevant shock tube to simulate blast TBI [171]. Similarly, no known published studies have chronically disrupted sleep prior to causing TBI. However, using the fluid percussion injury model to induce mild TBI in mice, Lim et al. demonstrated an inability for injured animals to maintain consistent waking during the dark (active) phase, a chronic fragmented sleep pattern with an increase in the number of wake to sleep transitions, and a decrease in sleep bout duration [172]. A similar study of moderate TBI in rats also reported a significant increase in wake to NREM transitions and a corresponding decrease in sleep/wake bout lengths that persisted chronically out to 29 days post-injury [173]. Both studies implicated deficits in the orexin system, which is consistent with the destabilization of waking during the active period [174]. These animal studies, amongst others, illustrate the similarities in excessive daytime sleepiness found between this model and civilian [175, 176] and military [177] cases of TBI. Integrating sleep measures into blast tube experiments, as well as other animal models of TBI, is critical to uncovering the link between neural injury and the sleep/wake system, as well as designing new treatment options for service members.

Similar to the TBI field, rodent models of stress and PTSD largely ignore the effects of pre-existing sleep dysregulation, or sleep alterations in response to stress exposure, on disorder severity or treatment outcome. A recent review authored by researchers at the Walter Reed Army Institute of Research (WRAIR) highlights the myriad methods used to model PTSD, as well as their respective strengths and weaknesses (Lowery-Gionta et al., unpublished data). Missing from these models are the integral ties to sleep and the role of pre-existing sleep disorders. Another timely review on the status of animal models of PTSD concluded that sleep alterations, amongst other metrics, should be included to increase translation of the results to human populations [178]. In fact, few studies have evaluated the impact on sleep architecture using stress or PTSD models, relative to the larger volume of literature. Initial publications using single bouts of social defeat stress in rats reported significant increases in acute NREM following conflict, regardless of winner or loser of the bout [179–182]. Sleep fragmentation was also increased 4 days after conflict, as compared to baseline and one day post-conflict, suggesting a time-dependent dysregulation of sleep architecture [182]. Limited to no changes in REM were found in these studies. A mouse study using a ten day chronic social defeat stress (CSDS) model found statistical increases in total REM time and number of bouts, as well as increases in NREM, during the ten days of CSDS, with only the increase in REM bouts remaining elevated following the protocol during the recovery phase, indicative of sleep fragmentation [183]. Pretreatment with a kappa opioid receptor antagonist prevented the increase in REM sleep bouts (reduced sleep fragmentation), alterations in circadian body temperature changes, as well as blocked alterations of the Clock gene mPer2 in mesolimbic structures, illustrating the benefit of integrating sleep markers into behavioral and treatment study designs.

Using the single-prolonged stress (SPS) model in rats, acute increases in total REM time and number of transitions to REM were reported in the active dark phase (lights off) on the day of SPS exposure, which then returned to baseline levels out to day 9 post-exposure [184]. The number of REM sleep bouts trended downward from days 5 to 9 during the sleep phase, whereas REM bout duration statistically increased, suggesting a time-evolving consolidation of REM sleep following exposure [184]. In another study using SPS in rats, an acute increase in waking was seen during the lights on sleep phase that coincided with statistically significant decreases in NREM and REM sleep [185]. Immediately following during the dark phase, both NREM and REM rebounded above baseline levels; REM sleep exhibited the largest increase, similar to findings from the Vanderheyden (2015) report. During the light phase, acute increases in body temperature were also reported during all phases of the sleep/wake cycle. Collectively, these studies highlight the ability of continuous EEG monitoring in rodent models of stress and PTSD to detect both immediate and delayed changes in sleep architecture that may offer clues to PTSD symptom development, and offer new translatable metrics to assess treatment efficacy.

#### Wireless telemetry

Sleep disruption paradigms (e.g. ECD) must be regularly employed prior to experimental manipulations to better simulate the military environment, whereas new wireless technology and software solutions are required to lower the entry burden into this field and promote more widespread adoption. Doing so will promote more ethologically relevant study designs with greater military relevance. These data will further our understanding of the role of sleep and sleep disruption in neural injury and recovery and guide development of more effective treatment regimens for service members.

Most sleep studies still rely on “tethered” EEG data acquisition systems that require animals to be removed from their homecages at set experimental intervals, placed in a recording chamber and connected to an amplifier via surgically implanted headmounts that allow access to brain and muscle activity. The majority of these tethered studies only take snapshots of an animals sleep/wake state during discrete time windows without accounting for changes that could be occurring during other periods of the day or night, missing large amounts of data in the process. Relying on specialized recording chambers limit study environments, particularly military relevant models and when using interactive studies such as the CSDS model of stress and depression. To circumvent these issues, fully implantable EEG telemetry systems are gaining traction across the research community (reviewed by Lundt et al. [186]). In-house military laboratories were an early adopter of the technology, and telemetry systems are routinely used to monitor recurring nerve-agent induced seizure activity [187–189], amongst other studies throughout the US Army Medical Command. These implants rely on small integrated batteries to wirelessly stream EEG, EMG and body temperature data to a nearby acquisition system, negating the need to physically connect the animals during each recording session. Importantly, continuous physiological sleep monitoring can be performed in the animals’ homecage, as well as in social environments [183, 190], without worrying about entangled wires. Unfortunately, their long-term use is constrained by battery life (~1.5 months, mouse; ~3 months, rat) and their physical size may also result in nuanced changes to animal behavior that can impact results in unanticipated ways [183]. That said, body temperature measurements streamed from these devices proved to be significantly altered in two of the previously discussed sleep studies [183, 185]; these findings would not be possible using traditional tethered EEG approaches and highlight the need for advanced technology to further understand sleeps’ role in brain disorders.

Dramatic reductions in miniaturized electronic platforms and advances in radio-frequency technology have the potential to revolutionize this field [191–194], as was demonstrated by wireless operation of implantable µLED’s for optogenetic studies [195]. Adapting this technology to include advanced electronics capable of wirelessly transmitting EEG, EMG, and body temperature would allow long-term continuous monitoring of sleep/wake states from baseline through recovery (Good and Lu et al., unpublished data). Advanced software capable of semi-automating the data processing and analysis pipeline will allow additional exploration of changes in EEG from these comprehensive datasets. Work is underway to integrate advanced EEG and sleep metrics into the predator exposure model of PTSD to understand the psychological ramifications of threat-to-life on sleep architecture [196]. Moving forward, our goal is to further integrate novel wireless technology into military relevant sleep disruption study designs for enhanced translation of results to service members.

### Sleep and performance

At present, there is a paradox in military regulations for optimal and minimal sleep amounts. The *Leader’s Guide to Soldier Health and Fitness* developed by the Office of the Army Surgeon General (ATP 6–22.5) recommends a Soldier sleep >7 h per night whenever possible. This regulation also recommends >9 h per night in preparation for episodes of inadequate sleep. In contrast, the minimum amount of sleep a US Army Soldier is required to achieve during field training exercises is >4 h per night per Army regulation TR 350–6, for example. A recent analysis of sleep amounts using wrist-worn actigraphy in armored (Army) battalions revealed the commonality of <5 h of sleep per night during training (at the National Training Center near Death Valley, CA) and deployment (Kuwait; Skeiky et al., unpublished data). In most cases, these Soldiers did not have consolidated sleep of <5 h per night but rather multiple bouts of <2 h. Twenty years before this study, the same group of researchers demonstrated a strong exponential relationship between nighttime sleep amounts and next-day performance on an artillery exercise at the same training location (National Training Center). For every hour of sleep lost, combat effectiveness degraded by 15–25% with Soldiers being at 15% total effectiveness with 4 hours per night (the current minimum per Army regulation TR 350-6) [33]. Reductions in combat effectiveness, as evidenced by cognitive performance [20] and marksmanship [197–199] decrements, as well as musculoskeletal symptoms [20], have been found following reduced or deprived sleep in recruits and trained warfighters. Whereas individual differences in how sleep loss impacts alertness and performance have been reported [200–202], only recently has the military begun exploring means to leverage these findings.

Sleep inertia is the transition period between sleeping and waking in which individuals display decrements in reaction performance and alertness, and can be seen as a reorganization of neural activity and connectivity patterns upon awakening from sleep states [203]. Rapid transitions from states of sleep to wakefulness across the nighttime and in the morning after a full night of sleep can degrade vigilance/alertness, as demonstrated by Balkin et al. in a military science laboratory [204], and are important aspects of human physiology to consider for military planning purposes. In fact, performance decrements resulting from sleep inertia experienced in the middle of a nighttime sleep episode after a few hours of sleep is similar to performance decrements under total sleep deprivation (>24 h extended wakefulness) [204]. Sleep inertia is maximal in the middle of the biological night when the circadian signal to sleep is highest, even if sleep requirements via the homeostatic system are met [205]. Sleep inertia is thought to be an operationally significant, yet overlooked problem. In deployed situations, service members are often rapidly awoken from sleep and must immediately attend to mission requirements that involve high levels of vigilance and life-or-death decision making (e.g. defensively returning fire following a base attack). To mitigate the effects of sleep inertia, military researchers at WRAIR demonstrated that performance decrements can be minimized through immediate caffeine intake upon waking [206]. It has also been found that sustained low-level caffeine consumption, combined with short naps, may be enough to stabilize performance and minimize sleep inertia under conditions of chronic sleep loss similar to that experienced in operating environments [207].

The ability to “bank sleep” to optimize performance under partial/total sleep deprivation, as well as enhance performance under normal conditions, is a burgeoning area of research that originated out of a military science laboratory (WRAIR) [72]. In Rupp et al., the authors showed that 10 h of time in bed (sleep extension) improved alertness and performance during subsequent sleep restriction (3 h per night) and recovery, as compared to individuals who slept their normal duration prior to sleep restriction. Mah et al. determined that when high-level collegiate basketball players extended their sleep duration by ~2 h (similar to [72], the players’ sprint times and shooting accuracies statistically improved, and their reaction times decreased, as measured using the psychomotor vigilance test (PVT) [208]. During partial sleep deprivation, napping has also been shown to reduce heart rate and boost short-term memory in athletes [209], as well as improve endurance in runners [210]. In an observational study of habitual sleep amounts in Reserve Officers’ Training Corps (ROTC) tactical athletes, measured through wrist-worn actigraphy, Ritland et al. determined that longer nighttime sleep durations correlated with increased motivation levels and better cognitive processing performance compared to military cadets with shorter sleep durations [211]; although other performance improvements were not found [211]. In another study, the same group experimentally extended sleep duration in ROTC tactical athletes by ~1.5 h and demonstrated statistical improvements in reaction time, athletic performance and motivation [212]. Importantly, some of these improvements persisted for up to four days following the end of sleep extension [212]. Collectively, these studies suggest that extending or “banking” sleep prior to operating missions in which sleep will be restricted could confer critical improvements in reaction time and performance for service members. Given the encouraging results from these studies, additional intervention-based studies of sleep extension are warranted, as the current state of the science regarding sleep duration and long-term health [213–215] and performance [216, 217] is heavily driven by large-scale observational/epidemiological studies of the general population.

#### Psychomotor vigilance test

Despite what occurs in field training environments, sleep research in military laboratories have shown that <5 h sleep per night is not sufficient to sustain performance [72, 218]. These same laboratories have also shown that countermeasures used to stabilize performance across sleep loss are ineffective after 3 days of <6 h sleep per night [218]. Broadly, the temporal relationship between sleep loss and performance decrements has been operationalized in laboratory studies using the PVT. The PVT is a computerized test of reaction time with a means to track inter-individual variability in neurobehavioral performance in real-time [219]. The test has high ecological reliability and validity [220] and translates very well to current military demands in numerous operating scenarios, including watch duty aboard US Navy ships [221]; it can be completed to monitor real-time vigilance in any environment, harsh or benign, with the availability of a smartphone-based version funded by the Army [222]. Current warfare, termed the multi-domain battle space, is one of cognition not attrition. Service members must endure long periods of sustained attention/vigilance during low-level, monotonous activities (e.g., driving in a convoy long distances from one operating base to another), knowing that the current weapon system of choice of our nation’s enemies are IED’s placed along roadsides or rocket-propelled grenades that can injure and kill numerous individuals at a moment’s notice. In this scenario, the PVT is ideal for monitoring ones reaction time and could serve as an early predictor of fatigue or loss of attention that could be detrimental to the entire unit.

With the PVT, researchers have been able to show temporal changes in performance across total sleep deprivation (forced wakefulness) and recovery sleep in order to determine “tipping points” in neurobehavioral decline [223]. In general, performance is stabilized across 24 h forced wakefulness [223] followed by a precipitous decline with >24 h of forced wakefulness [223, 224]. This performance decline can be protected, in part, by sleep extension prior to sleep loss [72], or having a PVT session coincide with a circadian-driven peak in alertness during increasing homeostatic pressure to sleep [224]. Finally, PVT performance is also predictive of the extent of recovery from total sleep deprivation [223].

The PVT has also been widely used to determine inter-individual variability in resiliency (or sensitivity) to sleep loss under both conditions of total sleep deprivation [223–225] and chronic sleep restriction [218, 226]. Whereas sleep extension prior to sleep loss helps to protect against performance decline, in general [72], inter-individual variability in resiliency to sleep loss is largely biologically regulated. Genetic association studies in humans revealed that select genotypes of an adenosine receptor (A2A) mediate the homeostatic drive to sleep [227], whereas select genotypes of dopamine metabolism (COMT) [228], and select genotypes of a clock gene (PER) known to mediate circadian timekeeping [229], can confer enhanced resiliency or sensitivity to varied conditions of sleep loss (total sleep deprivation, chronic sleep restriction; [230–235].

Resiliency to sleep loss can also be pharmacologically regulated. Caffeine’s half-life ranges from 3 to 7 h in adults [236], and can protect against performance decline during sleep loss in a dose- [237] and time-dependent manner [238]. Studies demonstrated that the ability of caffeine to sustain vigilance/alertness is genetically dependent. Habitual caffeine intake has been associated with reduced (self-rated) sleep quality in caffeine-sensitive individuals, but not in caffeine-insensitive individuals [239], regulated in part by single-nucleotide polymorphisms of the adenosine A2A receptor gene (ADORA2A; [239]. Further, ADORA2A polymorphisms not only mediate the extent of habitual caffeine use [240], but also mediate the extent of insomnia-like changes in the EEG induced by caffeine intake [231, 239]. Select genotypes of the adenosine receptor that additionally intersect with genotypes conferring sleep resiliency also confer enhanced (or reduced) sensitivity to the ability of caffeine to stabilize performance across sleep loss [231, 232]. However, it has been shown that the performance-enhancing effects of caffeine are ineffective for stabilizing performance under chronic sleep restriction [218] (<5 h sleep per night) which is the most common type of sleep loss that service members are exposed to (Choynowski et al., unpublished data) [241].

**Fig. 3:**
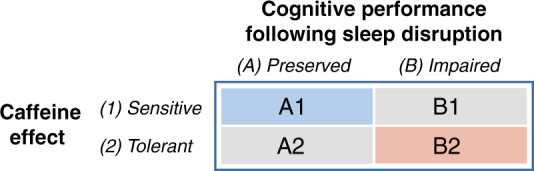
A working model of individual variation in the ability to perform in an operating environment under limited sleep conditions. The first principle is cognitive resilience to sleep disruption. Some individuals, due to biological modifiers, are able to preserve their level of vigilance and cognitive performance while sleep-deprived. The second principle is the degree of individual sensitivity to a caffeine countermeasure. This working model is thought to create a framework for group (unit-level) performance that can be optimized by stabilizing performance at the individual level and assigning duties accordingly

With this information, we have developed a working model to compartmentalize individual variability in sensitivity to sleep loss to determine “fitness for military duty” (Fig. 3). In our model, A1 individuals (blue box) would be most suited to perform in military operations. These individuals would have a genetic predisposition that allows their performance to be stabilized with sleep loss, but also sensitive to the performance-enhancing actions of caffeine. B2 individuals (red box) would be least suited to perform military operations. These individuals would have a genetic predisposition that degrades performance across sleep loss, but also linked to high caffeine tolerance. A2 and B1 individuals (gray boxes) would be moderately suited for military operations. There individuals would have stabilized performance with sleep loss (B1) but with a genetic trade-off of high caffeine tolerance (A2). Since individual variability in performance with sleep loss is high, this lends for the ability of military commanders to capitalize on genetic heterogeneity for the benefit of combat assignment and effectiveness. In fact, the Defense Advanced Research Projects Agency is currently focused on identifying novel genetic targets in order to eliminate subject bias for the candidate selection process, as well as recognizing persons who otherwise would not have been identified using high-throughput gene sequencing techniques. Through partnerships with academia, industry, and government, the intent is to use these rapid and non-invasive high-throughput sequencing techniques to determine fitness for duty and select mission assignment in these populations through a better understanding of genes regulating sleep and circadian synchrony.

#### Caffeine and sleep medications for fatigue management

Because the military has thrived on 24 h shift work operations, it has a rich history of stimulant and depressant use to maintain alertness or consolidate sleep, respectively. For instance, “Go” medications such as dextroamphetamine (Dexedrine) were once used to counter fatigue, but are no longer authorized (AFI 11–202, v3; AFSOCSUP 28 JULY 2017). However, it is widely known that military personnel over-consume caffeine (usually through energy drinks) in order to counteract the negative consequences of shift work, which in turn, amplifies acute stress responses [242]. Caffeine abuse and overuse during training and combat is a chief reason why caffeine-dosing strategies were recently developed by Army research laboratories [238, 243, 244] and implemented into regulations (ATP 6–22.5). Caffeine, taken at the right time and in correct dosage, has the ability to improve performance on certain tasks. For instance, 200 and 300 mg of caffeine improved marksmanship accuracy and sighting time, as compared to placebo and 100 mg, in sleep-deprived Navy SEAL trainees during Hell Week. The 200 mg dose group showed rapid improvements at 1 h, whereas the 300 mg dose suggested improvements in accuracy could last past 8 h based on salivary caffeine levels at that time point [198]. Another reason why caffeine is often abused and overused by military personnel is that only select units, such as aviation [245], are permitted access to pharmaceutical-grade wake-promoting agents (modafinil [100–200 mg; Trade name: Provigil] and armodafinil [150 mg; Trade name: Nuvigil]), despite modafinil stabilizing neurobehavioral performance during simulated shift work in a military science laboratory [246]. Whereas in the general population modafinil and armodafinil are prescribed to treat daytime sleepiness co-morbid with narcolepsy [247] and shift work disorder [248], a narcolepsy diagnosis requires medical discharge from the military.

Stimulants and depressants also have the ability to shift sleep/wake rhythms. Independent of genetic regulation of caffeine use and psychoactive effects, one study discovered that caffeine intake (200 mg; equivalent to a 16 ounce energy drink) 3 h before bedtime phase-delayed human endocrine (melatonin) rhythms by 40 min [249] with similar extrapolated findings in human cell culture (osteosarcoma U20S; [249]) and a mouse model [250]. However, beyond elevating daytime alertness, caffeine appears unable to entrain endocrine rhythms, as demonstrated in blind individuals [251]. In humans, caffeine has been shown to alter clock-driven melatonin release [249], but more research is warranted in this area in order develop better wake- and sleep-pharmacologic dosing strategies sensitive to individual and occupational demands.

The SCN is the central circadian pacemaker and has projections onto the mesolimbic system [252], and dopamine signaling in the ventral tegmental area is clock-driven [253, 254]. In animal models, GABA-derived depressants (alcohol) and monoamine-derived stimulants (cocaine) can block the ability of the circadian system to phase-shift to photic and non-photic stimuli [158, 255, 256]. The psychoactive effects of commonly prescribed sleep aids on human circadian rhythms are mild. Trazadone, a tricyclic antidepressant, and zolpidem, a non-benzodiazepine, have both been shown to marginally advance human circadian rhythms of core body temperature [257] and improve re-entrainment to shifted light-dark cycles [258] without affecting the circadian-controlled rhythm of REM sleep propensity [257].

Although research on the ability of vigilance-promoting nootropics and hypnotics to shorten/lengthen, phase-shift, and re-entrain human circadian-controlled rhythms is limited, it warrants further investigation within the context of military operations. Circadian desynchrony has recently been recognized as an operational threat in official military reports regarding future warfare, particularly when taking longer trans-meridian travel in the current geopolitical space into consideration, and more work is needed on means to counter this desynchronization.

## Conclusions

There are several factors contributing to the lack of sufficient, restorative sleep in the military, including combat operations, shift work, and comorbidities of psychiatric disorders and TBI in service members. Although real-time physiological and psychological health data in active duty personnel during deployed military operations is sparse, sleep disturbances, namely insomnia and OSA, have been broadly studied pre- and post-deployment and across biological sex in medical treatment facilities [5, 101, 102]. It is critical to understand these substrates and mechanisms - both physiological and psychological in nature -underlying comorbidity of sleep and psychiatric disorders as a path forward for sleep in the military. Understanding inter-individual variability in resiliency to sleep loss and stress can determine “fitness for military duty,” and can assist in placing a service member in an optimized military occupational specialty (MOS) and schedule that minimizes operational and personal risk and maximizes group performance and safety. The military is in the best position possible for changing cultural attitudes about sleep compared to civilian organizations. There are published guidelines all military leaders must recognize (The *Leader’s Guide to Soldier Health and Fitness* developed by the Office of the Army Surgeon General (ATP 6–22.5)) that provide strategies for achieving >7 h of sleep and maintaining vigilance during combat for the >400,000 ADMP. The military also possess machine learning-based tools to predict and counteract fatigue (through caffeine supplementation) in real-time (2B-Alert) [244]. The intent of these initiatives is to protect against immediate physiological and psychological decline during combat exposure in the short-term and optimize physiological and psychological health across one’s military career and into retirement.

### Future research directions

Long-term and comprehensive animal and human studies that include continuous sleep disruption designs are required to fully understand the physiological changes in neural circuitry following repeated circadian disruptions, and how these changes are intertwined with TBI and PTSD symptoms, amongst other neurological disorders. Studies of this nature must include numerous control groups that track the entire time-course of the experimental designs (and stages of the military life cycle), including matched healthy controls, circadian disrupted-only subjects and trauma exposed-only subjects; in order to properly dissect the role of pre-existing sleep disorders in disease severity and treatment outcome, as well as the restorative and protective effects of sleep following neurological insult and traumatic stress exposure. Using advanced technology capable of tracking physiology and behavior and integrating real-time countermeasures for many months in military personnel (e.g., Army’s 2B-alert) is critical to achieving these goals. Future research efforts will continue to focus on inter-individual variability, rather than group (unit-level) performance, in order to clearly identify individuals who are most sensitive and resilient to environmental stress and then use appropriately timed countermeasures in order to stabilize performance. Success of these comprehensive studies will additionally require consistent commitment from funding agencies, military organization leaders, academia and industry in order to realize the long-term benefits to help service members optimize their physiological and psychological health in the short-term (combat) and long-term (military career and retirement) from this approach. Lastly, it is our hope and anticipation that future iterations of animal models such as the CSDS [259], single prolonged stress, and blast TBI protocols, amongst other models of neurological disorders [178], will integrate sleep more readily into study designs.

## Funding and disclosure

CHG is supported by a Laboratory University Collaboration Initiative (LUCI) Fellowship sponsored by the Office of the Under Secretary of Defense for Research & Engineering. AJB and VFC are supported by the Military Operational Medicine Research Program. The opinions or assertions contained herein are the private views of the authors, and are not to be construed as official, or as reflecting true views of the Department of the Army or the Department of Defense. The authors declare no competing interests.
